# Why the Energy Landscape of Barnase Is Hierarchical

**DOI:** 10.3389/fmolb.2018.00115

**Published:** 2018-12-20

**Authors:** Maya J. Pandya, Stefanie Schiffers, Andrea M. Hounslow, Nicola J. Baxter, Mike P. Williamson

**Affiliations:** Department of Molecular Biology and Biotechnology, University of Sheffield, Sheffield, United Kingdom

**Keywords:** protein dynamics, nuclear magnetic resonance (NMR), biophysics, structural biology, molecular dynamics, conformational selection, relaxation dispersion

## Abstract

We have used NMR and computational methods to characterize the dynamics of the ribonuclease barnase over a wide range of timescales in free and inhibitor-bound states. Using temperature- and denaturant-dependent measurements of chemical shift, we show that barnase undergoes frequent and highly populated hinge bending. Using relaxation dispersion, we characterize a slower and less populated motion with a rate of 750 ± 200 s^−1^, involving residues around the lip of the active site, which occurs in both free and bound states and therefore suggests conformational selection. Normal mode calculations characterize correlated hinge bending motions on a very rapid timescale. These three measurements are combined with previous measurements and molecular dynamics calculations on barnase to characterize its dynamic landscape on timescales from picoseconds to milliseconds and length scales from 0.1 to 2.5 nm. We show that barnase has two different large-scale fluctuations: one on a timescale of 10^−9^−10^−6^ s that has no free energy barrier and is a hinge bending that is determined by the architecture of the protein; and one on a timescale of milliseconds (i.e., 750 s^−1^) that has a significant free energy barrier and starts from a partially hinge-bent conformation. These two motions can be described as hierarchical, in that the more highly populated faster motion provides a platform for the slower (less probable) motion. The implications are discussed. The use of temperature and denaturant is suggested as a simple and general way to characterize motions on the intermediate ns-μs timescale.

## Introduction

It is generally accepted that the dynamic landscape of a protein (that is, the set of conformations it can adopt, and the rates at which it can move between them) is critical for its function (Hammes-Schiffer and Benkovic, 2006). Proteins all have to bind to other molecules, and thus in general have a free (often called “open”) and a bound (often called “closed”) conformation (Ma and Nussinov, 2010). Exchange between these two states can be stimulated by ligand binding [“induced fit”: (Sullivan and Holyoak, 2008)] or can occur in the absence of ligand (“conformational selection”), the latter of which appears to be more common for enzymes under physiological conditions (Beach et al., 2005; Eisenmesser et al., 2005; Boehr et al., 2006; Aleksandrov and Simonson, 2010), though often the detailed conformational changes appear to require some combination of these two extremes (Okazaki and Takada, 2008; Zhuravlev and Papoian, 2010; Williamson, 2011; Vogt and Di Cera, 2012).

The energy for protein motion in solution is thermal in origin (Frauenfelder et al., 2009). Thermal motion of the bulk solvent leads to rapid local and uncorrelated motions in proteins, on a femtosecond/picosecond timescale (Frauenfelder et al., 2009). These very small and very rapid motions can lead to localized bond rotations, on picosecond/nanosecond timescales. Occasionally, these local motions are synchronous, leading to larger scale and slower correlated motion (Williamson, 2011). It is important to recognize that even the slower transitions still involve atomic movement on a rapid timescale; the more highly correlated motions just occur infrequently and with low probability, and for that reason can be called “slower”. Such motions are difficult to characterize experimentally, but can be described using molecular dynamics and other computational techniques (Gao et al., 2006; Ma and Nussinov, 2010; Ramanathan and Agarwal, 2011). One such efficient and informative technique is normal mode (elastic network) calculations (Zhuravlev and Papoian, 2010), in which the protein is represented as a set of masses connected by harmonic springs, and the resulting coupled differential equations are solved to obtain low-frequency eigenmodes. Normal mode calculations present a more direct picture of domain movement than do molecular dynamics simulations, because they are free from the random thermal motions. Such calculations are fast and widely used (Bahar et al., 2010), and the motions have been shown to be largely determined by the architecture of the protein (Zhuravlev and Papoian, 2010; Bahar et al., 2015), a conclusion supported by the conservation of flexibility in homologs (Gagné et al., 2012). An examination of the lowest energy normal modes shows that, in agreement with the expectation above, they generally involve movements of rigid domains around small hinge regions.

Most enzymes have catalytic turnover rates in the range 10^2^–10^5^ s^−1^ (Wolfenden and Snider, 2001), and it is generally agreed that these rates are determined not by the chemical reaction rate itself but by the rate at which the enzyme structure alters to allow substrate to bind and product to leave (Agarwal et al., 2002; Hammes-Schiffer and Benkovic, 2006; Williamson, 2011). Thus, protein rearrangements at rates in the millisecond range are widely seen as crucial to enzyme function. This therefore leaves a big problem: how do the local uncorrelated fluctuations described above, typically at 10^9^ s^−1^ or faster, get channeled into large-scale correlated fluctuations occurring at around 1,000 s^−1^? Also, how are the different fluctuations *coupled*, and how does one motion *facilitate* another? A typical answer from the literature is that “the solvent is coupled to the flexible surface loops, which eventually transfer the kinetic energy to the active site through the conserved network interaction” (Ramanathan and Agarwal, 2011). This is fine as far as it goes, although the physical mechanism for achieving it remains unclear, especially as in some models, the network need not be composed of contiguous residues (Hammes-Schiffer and Benkovic, 2006; Wrabl et al., 2011).

Enzymes require a conformational change from a low-activity ground state, approximately the ground state as seen by crystallography or NMR, to a higher activity excited state, both to bind the substrate effectively and shield it from water, and also to allow rapid movement of substrate and products on and off the enzyme (Hammes et al., 2011). These two states are often equated to the free and bound states described above, although experimental verification is difficult. Even more unclear is the extent to which enzyme motions might directly drive the reactants over the transition state (Hammes et al., 2011; Glowacki et al., 2012), a concept sometimes described as a promoting vibration (Antoniou and Schwartz, 2011; Hay and Scrutton, 2012). In this paper, we do not characterize the chemical reaction itself, and therefore do not consider the question of promoting vibrations further.

Enzymes have evolved structures that enable them to adopt different conformations linked to ligand binding. These conformational changes have been described as *promoting motions* (Agarwal et al., 2002; Loveridge et al., 2011): motions that generate a reaction-ready or near-attack conformation (Lightstone and Bruice, 1996), after which the chemical reaction itself occurs rapidly (Lodola et al., 2007; Watt et al., 2007; Antoniou and Schwartz, 2011; Doshi et al., 2012). Note that these are quite different from the promoting vibrations discussed above, which are much faster and directly push the substrate over the transition state. Henzler-Wildman et al. (2007) popularized the idea that several different promoting motions could occur *hierarchically*, with one motion facilitating another. This is an attractive idea but it has proved difficult to study experimentally: in particular, experimental techniques for characterizing intermediate rate motions on the nanosecond/microsecond timescale have proved elusive (Lange et al., 2008; Hansen et al., 2009; McDonald et al., 2013). There is thus a gap in our current understanding: how can motions on the nanosecond scale “facilitate” conformational changes on the millisecond scale, roughly one million times slower, and thus what does it mean to describe two motions as hierarchical? The aim of this paper is to characterize two different motions in barnase; show how the faster one makes the slower one possible; and discuss the consequences, which provide a framework to understand the term *hierarchical*.

The ribonuclease barnase is a well-studied 12 kDa enzyme, and is thus a good model system for studying the relationship between dynamics and function. Its catalytic mechanism, folding and ligand binding are well-characterized. There are a number of crystal structures of free barnase and of its complexes, in particular a high-resolution structure bound to the deoxynucleotide inhibitor d(CGAC) (Buckle and Fersht, 1994), showing a small but well-defined closure of the enzyme around the inhibitor. There have been studies of its dynamics carried out using molecular dynamics (MD) (Nolde et al., 2002; Giraldo et al., 2004; Zhuravleva et al., 2007), and using ^15^N and methyl group NMR relaxation (Sahu et al., 2000; Zhuravleva et al., 2007). These have shown that the enzyme has three rigid hydrophobic cores, with the major motion being a hinge-bending of the β-sheet, which lies across the center of the enzyme. Because the active site lies over the top of the β-sheet, the hinge bending leads to a small closure of the active site loops over the substrate.

Here we use two NMR techniques to probe the dynamics of barnase in more detail: a technique that we first described several years ago, which involves measuring the effect of temperature on the chemical shift of amide groups (Baxter et al., 1998; Williamson, 2003; Tunnicliffe et al., 2005), and is developed further here, and relaxation dispersion, which provides information on collective motions in the millisecond range (Korzhnev et al., 2004). We characterize two large-scale motions in barnase. The higher frequency motion is characterized using temperature-dependent shift changes, consists of hinge bending across the β-sheet, occurring at a rate faster than 10^6^ s^−1^, and is populated to at least 5%. The second motion involves residues around the lip of the active site in addition to the β-sheet hinge, is populated at any one time to no more than 2%, and occurs at a much slower rate of 750 ± 200 s^−1^, with a much larger free energy barrier. We show that the second motion is *facilitated* by the first, in that it occurs most efficiently from a partially hinge-closed state that is produced by the first motion: the two motions are therefore categorized as hierarchical. Hence, we provide an unusually detailed description of two coupled hierarchical motions, with one facilitating the other. The sum effect of both motions is a conformational exchange from the open ground state to a closed local energy minimum, to generate the reaction-ready closed conformation.

## Materials and Methods

### Materials

Barnase H102A mutant was expressed in *Escherichia coli* strain M15 and purified using a two-stage ion exchange chromatography protocol shown to produce protein free from bound nucleotide (Cioffi et al., 2009). The inhibitor d(CGAC) was purchased from Metabion International AG (Martinsried, Germany) and used without further purification. It was added to the protein to produce an ~4.5-fold excess of ligand over protein. Given the dissociation constant of 49 μM under our conditions (Cioffi et al., 2009), this represents essentially fully bound enzyme.

### NMR

Chemical shift assignments for free and bound barnase were taken from BioMagResBank entries 16,169 and 16,171, respectively. For the temperature dependence, shifts were measured from 2D HSQC spectra every 5°C from 288 to 313 K, and peak positions were picked manually in the program FELIX (Felix NMR, Inc.) using in-house routines, and analyzed for curvature by fitting to a quadratic equation. The second-order coefficient of the quadratic was used as the measure of curvature. This procedure was repeated at guanidinium hydrochloride concentrations ([Gdm]) of 0.4, 0.8, 1.2, and 1.6 M. The second-order coefficients were then plotted as a function of [Gdm] and again fitted to a quadratic. Residues for which the fits were poor (as judged by eye and also by analysis of χ^2^) were discarded. The fitting returned means and standard deviations of slope and curvature for each residue. These were taken to be significantly different from zero when the signs of (mean ± 2 × standard deviation) were the same, i.e., they did not span zero. Ring current effects from nucleotide binding on ^15^N chemical shifts were calculated from the crystal structure of barnase bound to d(CGAC), 1brn, using the program *total* (Williamson and Asakura, 1993). Ring current intensity factors for nucleotides were taken from (Case, 1995). In particular, we assume that the ring-current induced chemical shift change of a ^15^N nucleus is the same in ppm as that for a proton in the same location (Blanchard et al., 1997).

Relaxation dispersion (RD) experiments were carried out using 1 mM ^15^N-labeled barnase H102A, pH 5.8 in 50 mM sodium acetate buffer, 0.02% sodium azide, 25°C. Initial experiments were carried out in different buffers, showing that phosphate binds in the active site and makes the magnitude of relaxation dispersion profiles much smaller. Tris also gives less satisfactory results. Varying the protein concentration gave no observable changes in chemical shifts or relaxation rates, implying that barnase remains monomeric under these conditions. Experiments were carried out in Shigemi tubes (CortecNet, Paris) to minimize experimental artifacts due to convection and evaporation, on Bruker Avance DRX 800 and DRX 600 spectrometers, the 600 being equipped with a cryoprobe. Temperatures were calibrated using perdeuterated methanol (Findeisen et al., 2007). ^15^N Carr-Purcell-Meiboom-Gill (CPMG) RD data were collected as described (Loria et al., 1999), modified by duty-cycle heating compensation and a pre-scan saturation sequence (Yip and Zuiderweg, 2005; Long et al., 2010). Interleaving of spectra at the scan level was tried but gave worse results. A series of 15 2D spectra was collected with the CPMG field ν_CPMG_ covering a range of 50 to 750 Hz, in random order, with repeat experiments performed at 300 and 500 Hz (at 600 MHz) or 400 Hz (at 800 MHz) and a reference spectrum obtained by omitting the CPMG period in the pulse sequence and doubling the length of the compensation block. The relaxation delay, *T*_CPMG_, was 40 ms, and the recycle delay was 2.5 s. To reduce off-resonance effects, spectra were obtained using two different ^15^N offsets for each delay.

Spectra were processed and analyzed using Topspin (Bruker Biospin) and FELIX. The effective transverse relaxation rate, *R*${}_{2}^{\text{eff}}$, was calculated for each ν_CPMG_ value from cross-peak intensities:

$$\begin{array}{l} R_{2}^{eff}\left(\nu_{CPMG}\right)=-\frac{1}{T_{CPMG}}ln\left(\frac{I_{CPMG}}{I_{0}}\right) \end{array}$$

where ν_CPMG_ = 1/(2τ_CPMG_) and τ_CPMG_ is the delay between successive refocusing pulses in the CPMG pulse train, *I*_CPMG_ is the peak intensity obtained with a given CPMG spacing and *I*_0_ is the intensity of a peak when the relaxation delay is omitted. 19 residues were excluded from the analysis because of overlap or weak intensity at one or both fields. RD data were fitted to two-site exchange with the Levenberg-Marquardt non-linear least-squares algorithm using the complete modified Bloch-McConnell equations (Korzhnev et al., 2004; Demers and Mittermaier, 2009) by explicit numerical modeling, using the *numpy* and *scipy* python modules for the evaluation of matrix exponentials. Residues were selected for fitting when the magnitude of the dispersion, max(*R*${}_{2}^{\text{eff}}$)–min(*R*${}_{2}^{\text{eff}}$), was larger than 1.0 s^−1^ at both fields (free barnase), which defined residues 36, 39, 42, 43, 56, 58, 83, 85, 86, 101, and 106. Following trials that showed that all residues fitted (within error) to a common exchange rate, data were fitted simultaneously for all signals (Long et al., 2010). Residues 36, 39, 43, 56, and 58 were subsequently excluded from the analysis because the errors in the fitting as judged by χ^2^ values were too large (Demers and Mittermaier, 2009). Data were fitted using different residues and fixing different relaxation rates or exchange rates, to check for consistency in the fitting. In the final fitting, *R*_2_ rates in the absence of exchange were assumed to be identical at both sites, but were allowed to vary independently for each residue. Exchange was shown to be close to the fast limit from the fitted value of the exchange rate, from the fact that the effects weaken as the temperature is raised, and from calculation of the exchange rate parameter α as described (Millet et al., 2000), which has a value of 1.6 ± 0.1. In the final fitting, data were fitted simultaneously for both field strengths, using a common exchange rate and population ratio (and thus common forward and back rates) for all residues, and *R*_2_ rates in the absence of exchange that were the same in both free and bound states, but different for each residue. Fits were also carried out by fixing the on-rate at different values, or by fixing the off-rate, to check for a robust search minimum. The optimized fit (to all data at both field strengths) had a χ^2^ value of 537, with *R*_2_ experimental errors estimated at 0.2 s^−1^ based on analysis of experimental errors. This χ^2^ value is rather large, implying the presence of some systematic errors, likely the presence of additional slow exchange processes for a small number of residues, as described in section Results.

### Computation

Normal modes were calculated from the PDB files 1a2p (molecule a) (Martin et al., 1999) and 1brn (molecule l) (Buckle and Fersht, 1994) [the highest resolution structures of free and d(CGAC)-bound barnase, respectively], using NOMAD-Ref (Delarue and Sanejouand, 2002; Lindahl et al., 2006) with the default parameters (5 Å distance weight parameter and 10 Å elastic network model cutoff), using all heavy atoms. For each mode, the structural changes were rescaled to give an average RMSD of 1.0 Å over the full range. Structures were analyzed using Pymol.

## Results

### Fast Fluctuations Determined by Temperature-Dependent Curvature

A curved temperature dependence of NMR chemical shifts indicates the presence of one or more alternative states, exchanging faster than the chemical shift timescale (Williamson, 2003; Tunnicliffe et al., 2005; Mohan and Hosur, 2008; Srivastava and Chary, 2011). However, curvature of temperature dependence provides an unreliable guide to the location of alternative states, because the amount of curvature is affected both by the chemical shift difference between the two states, and by the free energy difference between the two states at each temperature, which in general are not known. These effects mean that some residues that sample alternative states show no apparent curvature. To remedy this problem, we have shown that curvature is affected progressively by the addition of guanidinium hydrochloride (Gdm), which destabilizes the ground state more than it destabilizes alternative states (Tunnicliffe et al., 2005). This makes the experiment a clearer guide to the presence of alternative states. Here, we have analyzed the change in curvature in a new and more systematic way, described below, which gives a much clearer and more reliable guide to the locations of rapidly exchanging conformational states. For simplicity hereafter, we describe this technique as *Curvature of amide proton shifts induced by denaturant* (CAPSID).

**Figure 1:**
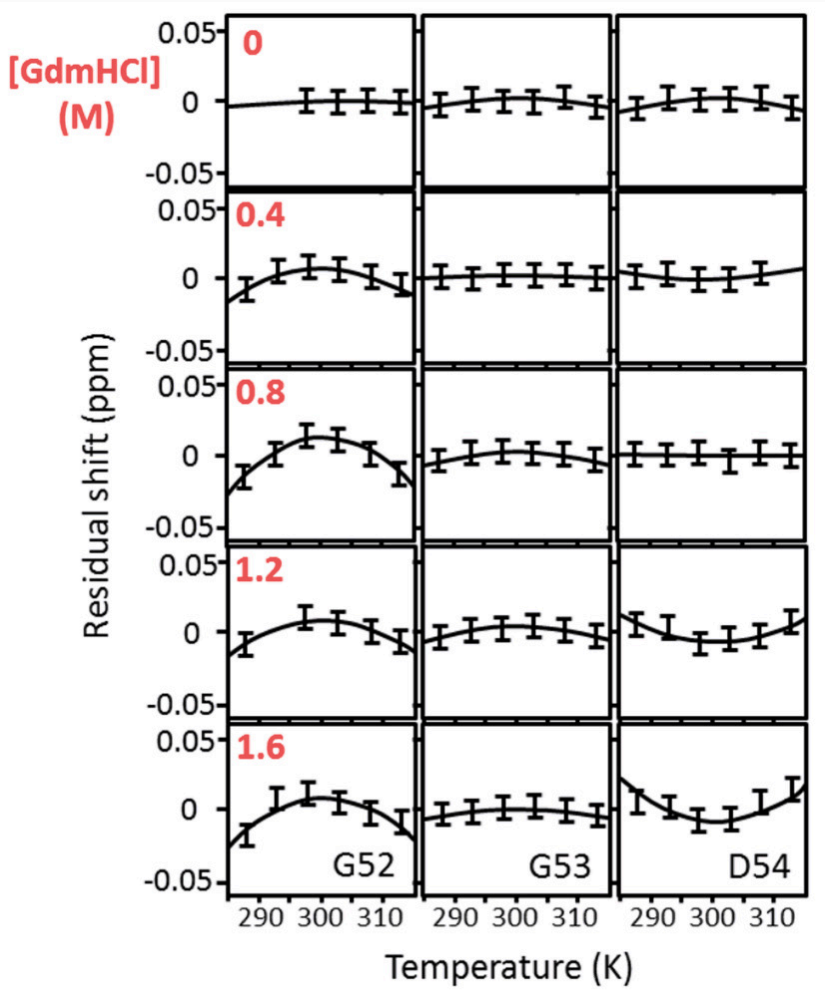
Temperature-dependent chemical shifts of Gly52, Gly53, and Asp54 of barnase H102A at 5° intervals over the range 288–313 K, and at GdmHCl concentrations of 0, 0.4, 0.8, 1.2, and 1.6 M. The data are for the complex of barnase with d(CGAC). Some missing data are due to peak overlap. The linear temperature dependences (which are in the region of −4.5 ppb/degree) have been subtracted to make the curvatures more obvious. The size of the error bars represents the approximate error in measurement of peak position (±0.0075 ppm).

**Figure 2:**
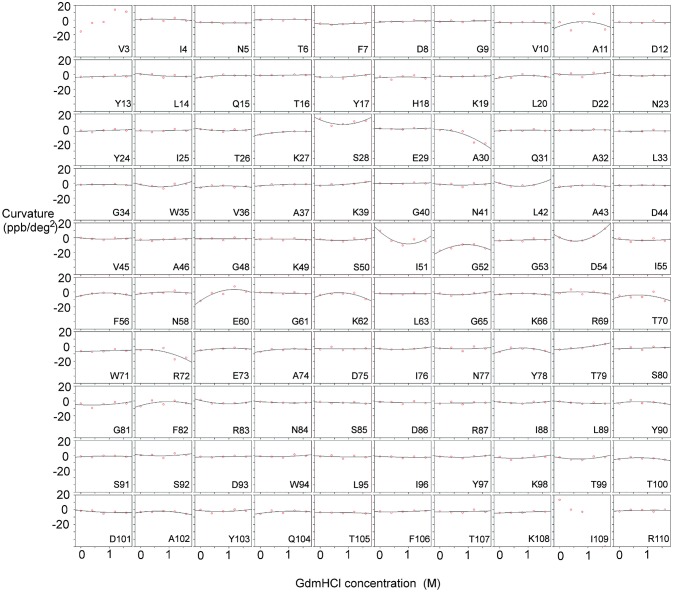
Temperature-dependent curvature for each residue plotted against GdmHCl concentration, for free barnase. Note that zero curvature corresponds to a flat line through zero, near the top of each plot. The data for each residue are fitted to a parabola. For two residues (Val3 and Ile109) the data could not be fitted reliably. Some residues (for example Ala11 and Tyr78) have a markedly curved fit, but the scatter in the data means that the fitted curve is not significantly different from zero (see section Materials and Methods). Other residues (for example Asn5 and Phe7) have fits that are close to zero, but on testing turn out to be significantly different from zero.

We measured the temperature dependence of the chemical shift of amide protons in barnase over the range 288–313 K, and fitted this to a quadratic equation (*y* = *a* + *bx* + *cx*^2^) (Figure 1). The curvature of the temperature dependence is given by the fitted value of *c*. We then plotted *c* against [Gdm] for a range of Gdm concentrations up to 1.6 M, which remains well-beneath the concentration required for denaturation of barnase (Clarke and Fersht, 1993). This plot makes the chemical shift curvature much more obvious, particularly when curvature in the absence of Gdm is small (Figure 2). For most residues, *c* is very close to zero, and there is no significant dependence of *c* on [Gdm] (as defined in section Materials and Methods). However, for 16 residues (from a total of 100 that could be followed across all spectra), a well-defined dependence could be observed (residues 5, 7, 27, 28, 30, 51, 52, 54, 60, 71, 72, 73, 74, 100, 101, and 102). The shapes of these dependences vary (concave, convex or flat but non-zero): in general, a concave shape shows that the excited state has a chemical shift greater (downfield) than the ground state whereas a convex shape shows that the excited state has a smaller chemical shift. Thus, a monotonic Gdm-dependent change in curvature tells us that the residue has a low-lying excited state, within ~10 kJ mol^−1^ of the ground state (Williamson, 2003), with the sign of the change telling us the direction of the shift difference.

**Figure 3:**
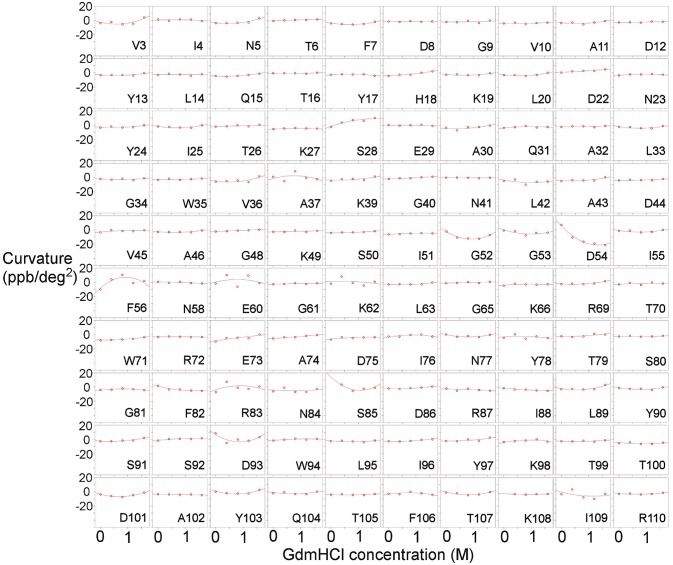
Temperature-dependent curvature for each residue plotted against GdmHCl concentration, for the complex of barnase with d(CGAC). See legend for Figure 2 for other details.

Measurements were also carried out for barnase bound to d(CGAC) (Figure 3). Of the 16 residues found to have Gdm dependence in free barnase (out of 110 residues total), 14 also have Gdm dependence in the bound state (all except 60 and 72). There are an additional two residues (53 and 85) observed in the bound state but not seen in the free state. The close correspondence between the two sets of residues suggests that the rapid fluctuation occurring in free barnase is also occurring in bound barnase, in a very similar location. We have no direct measure of the timescale of this fluctuation, except we can conclude that it is much faster than 10^5^ s^−1^ (because it does not give rise to line broadening) and slower than the processes observed by backbone (Sahu et al., 2000) and methyl (Zhuravleva et al., 2007) relaxation, which are ~10^11^ s^−1^.

The close similarity of the affected residues in free and bound states provides strong evidence that free and bound barnase are sampling the same range of motion, and therefore that the rapid low-energy fluctuations in free and bound barnase cover a similar range of conformational states: in other words, both free and bound barnase are capable of similar large-scale movements, implying similar shapes of energy landscape for free and bound.

### Millisecond Motions in Free Barnase

**Figure 4:**
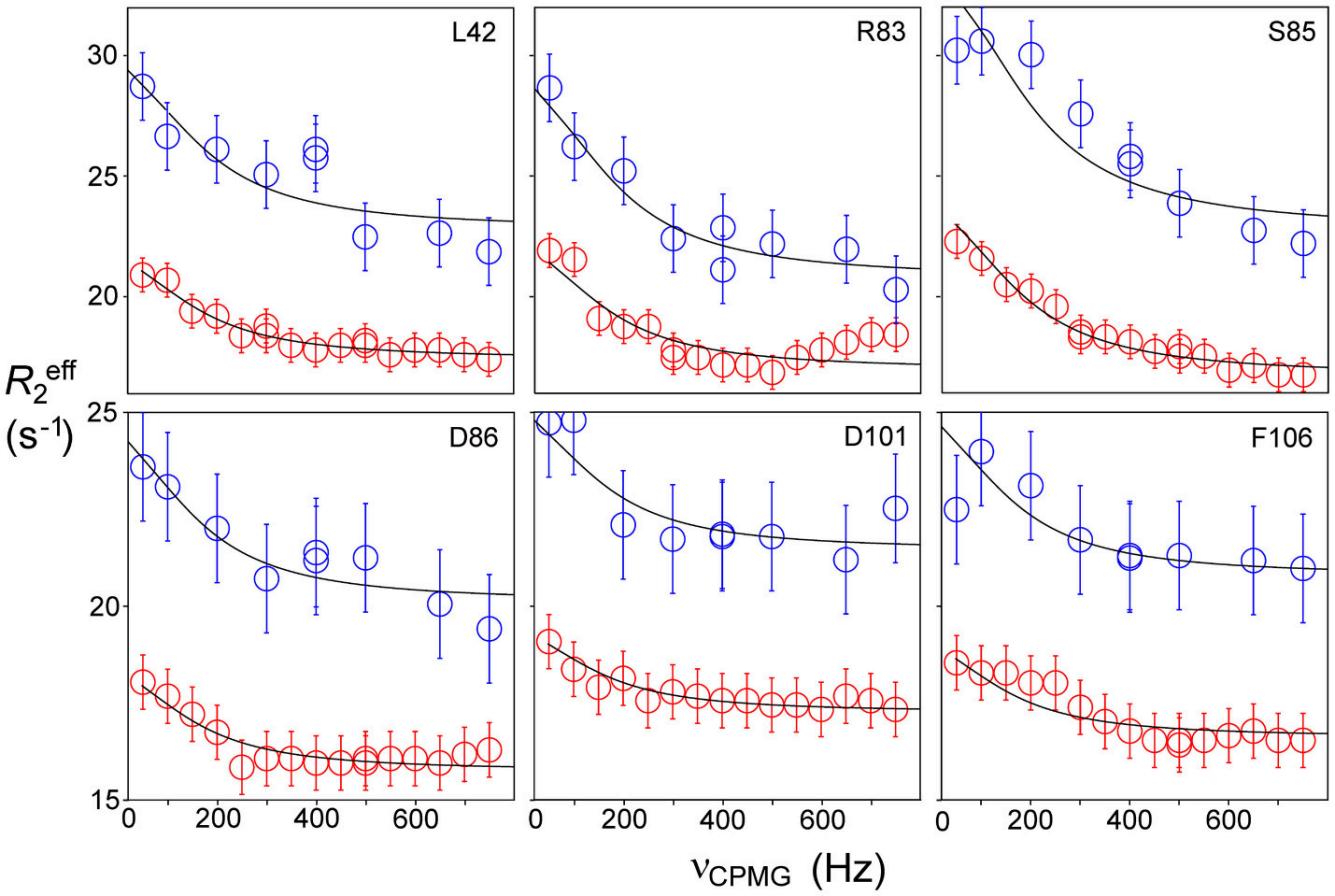
Relaxation dispersion profiles for free barnase at 298 K. Data are shown for the six residues showing the largest measurable effects. Five other residues (36, 39, 43, 56, and 58) also have clear profiles at both fields, but smaller than those shown here, while L14 is clearly exchanging but could not be measured accurately because of overlap. These residues are shown on the protein structure in Figure 9 below. Experimental data are represented by red and blue circles at 600 and 800 MHz, respectively. Errors in measured relaxation rates are ~0.7 s^−1^ at 600 MHz and 1.4 s^−1^ at 800 MHz.

**Figure 5:**
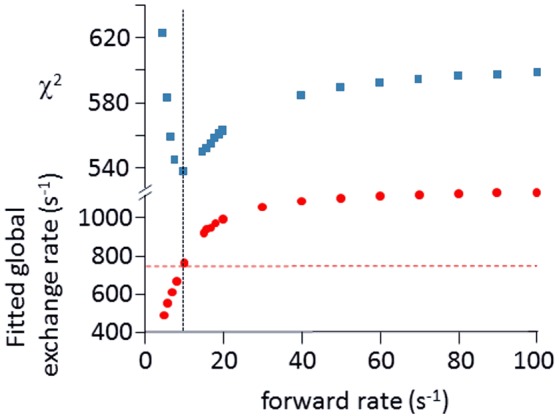
Estimation of forward rate for free barnase. The data shown in Figure 4 at both field strengths were fitted to common forward and back rates (or equivalently, a common global exchange rate and population ratio), with residue-specific values for the ^15^N chemical shift differences. The best fit was 750 ± 200 s^−1^ for the global exchange rate (indicated by the dashed horizontal line in the bottom panel), with a forward rate of 10 s^−1^ (vertical dashed line, bottom panel). As an alternative approach, the forward rate was fixed to various values, and the data were then re-fitted (bottom panel, filled circles). The quality of the fit is indicated by the χ^2^ values shown in the upper panel, which have a minimum at the same forward rate of 10 s^−1^, demonstrating the consistency between the two approaches.

Relaxation dispersion (RD) experiments were carried out at a range of field strengths and temperatures, with the best data being obtained at 298 K. A number of residues show RD profiles, indicating that they are involved in slow (millisecond) conformational exchange. Data for the resolved signals showing the largest changes in *R*${}_{2}^{\text{eff}}$ are shown in Figure 4. The data at 600 MHz have smaller errors than those at 800 MHz. When fitted individually to a standard two-state model, all residues fitted to similar exchange rates, although with large errors; they were therefore constrained to fit simultaneously to the same overall exchange rate, which improves the reliability of the fit. Exchange is close to the fast limit, as indicated by an < α> value of 1.6 ± 0.1 (Millet et al., 2000). In this limit, fitting at a single field strength only provides a value for the product p_A_p${}_{\text{B}}\Delta \omega^{2}$, where p_A_, p_B_ are the fractional populations of the two exchanging states and Δω is the difference in ^15^N chemical shift. Fitting at two fields provides tighter limits, because Δω is proportional to field strength, although the poorer quality of data at 800 MHz limits the reliability of the fit (Kovrigin et al., 2006). Therefore, the data in Figure 4 were fitted to a global exchange rate (the sum of on and off rates) and residue-specific values of p_A_p${}_{\text{B}}\Delta \omega^{2}$, with the additional constraint that the value of p_A_p_B_ should be the same for all residues. As seen in Figure 4, this assumption fits the data reasonably well, giving a global exchange rate of 750 ± 200 s^−1^, although the data for S85 (and possibly also F106) at 800 MHz suggest an additional slower exchange process may be affecting these residues. The global exchange rate is the sum of the forward and back rates, the back rate being much larger than the forward rate. The forward exchange rate obtained from this analysis is 10 s^−1^ (Figure 5, vertical dashed line), although it is subject to considerable error. It can be best estimated by fixing it to different values and re-fitting the data (Figure 5). This process results in a reasonably well-determined best-fit forward rate of 10 ± 5 s^−1^.

**Figure 6:**
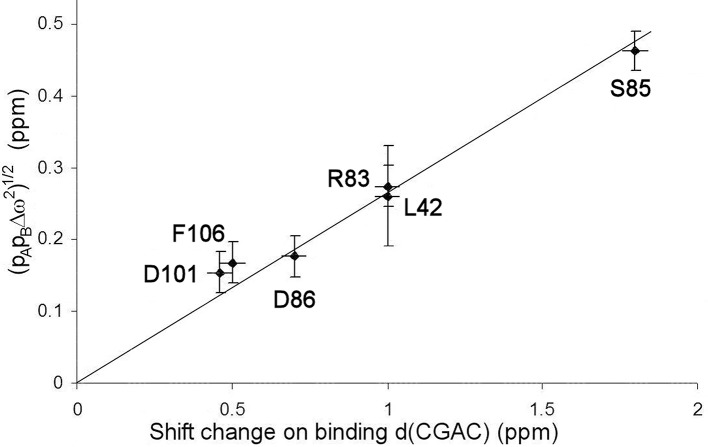
Comparison of chemical shift changes calculated from fitting to relaxation dispersion data for free barnase [expressed as (p_A_p${}_{\text{B}}\Delta \omega^{2}$)^1/2^], with chemical shift changes observed on adding d(CGAC) to barnase. The error in the binding shift change of Arg83 (in the center of the plot) is ~1 ppm because it is expected to hydrogen bond to nucleotide in the bound state (Buckle and Fersht, 1994), which leads to large and rather unpredictable shift changes (Xu and Case, 2002). The residues used for the fitting are those shown in Figure 4.

The excited state identified here is, by definition, in a local energy minimum close in energy to the ground state. The residues that define the excited state are found to line the active site (see section Discussion below). We therefore hypothesized that the conformational exchange occurring is between a ground state “open” conformation and an excited state “closed” conformation that closely resembles the conformation when substrate or competitive inhibitor is bound: in other words, that this is an example of conformational selection, and therefore that the ^15^N chemical shift changes derived from the RD fits should match the shift changes seen experimentally on going between free and bound states (with suitable allowance made for chemical shift changes arising from direct interaction), as seen in RD studies on other enzymes (Beach et al., 2005; Boehr et al., 2006). Figure 6 shows that the shifts derived from the fit shown in Figure 4 do indeed match the experimental shifts, after correction for ring current shifts (of which the largest is an effect of 0.15 ppm at Ser85). Thus, the hypothesis is confirmed. We therefore used the slope of the correlation line in Figure 6 to obtain the product p_A_p_B_, which is 0.02, implying a forward rate of 15 ± 5 s^−1^. The excellent agreement with the direct fitting above further strengthens the hypothesis of conformational selection. A forward rate of 15 s^−1^ implies a population of 2% of molecules in the excited state. We note that the fitted ^15^N chemical shift changes are small, and are consistent with the small structural change in the barnase crystal structures between free and d(CGAC)-bound.

It is not a surprise to find barnase presenting evidence of conformational selection in ligand binding. Similar properties have been found for other small enzymes with fairly unhindered closed states, including dihydrofolate reductase (Boehr et al., 2006), ribonuclease (Beach et al., 2005), and cyclophilin A (Eisenmesser et al., 2005).

### Millisecond Motions in Bound Barnase

**Figure 7:**
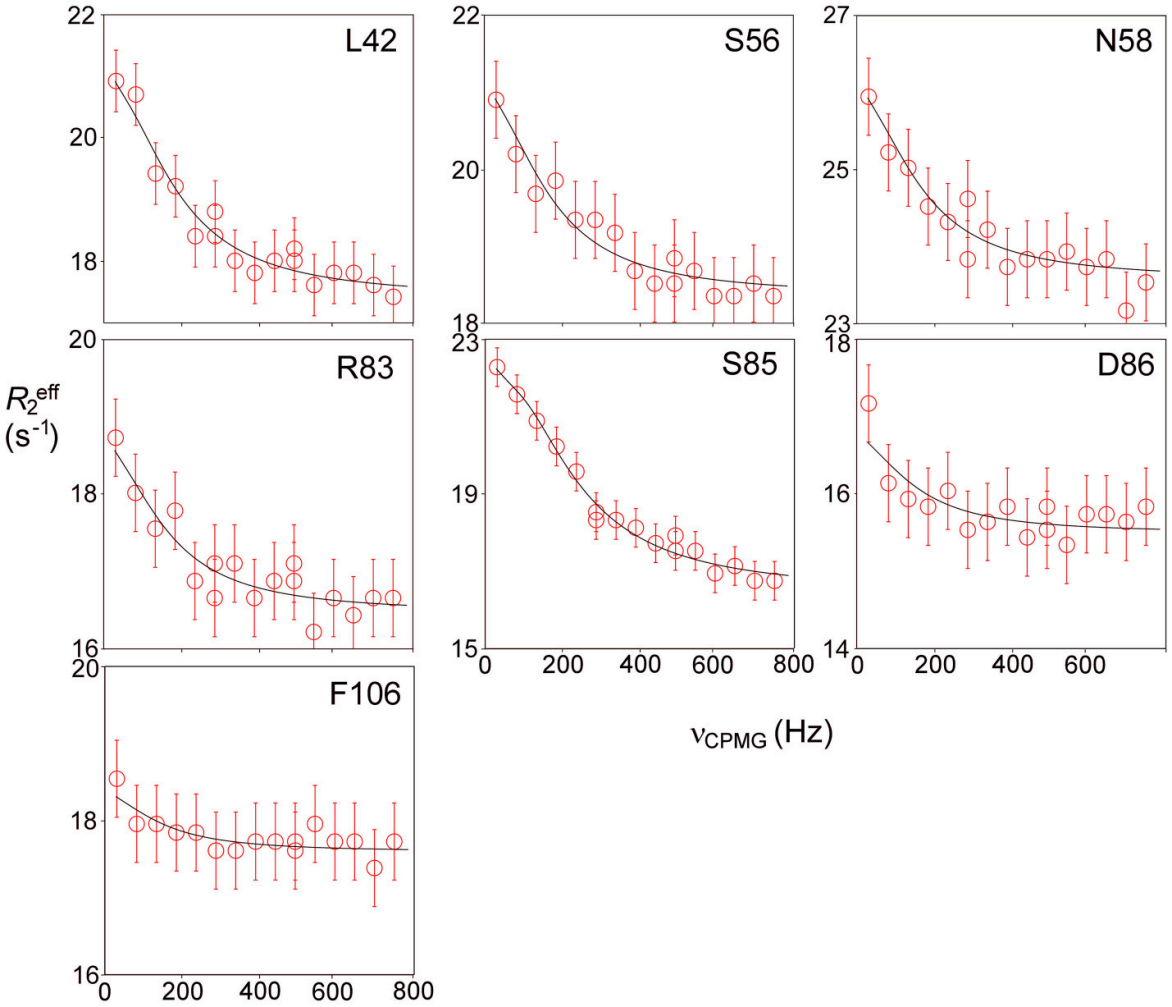
Relaxation dispersion data for barnase bound to d(CGAC) at 298 K. Data are shown for the seven residues showing the largest effects at 600 MHz. Experimental data are represented by the circles, with errors of ±0.5 Hz. Data were fitted to a global forward and backward rate, with individual chemical shift differences fixed at the changes observed experimentally between bound and free, corrected for ring current shifts.

**Figure 8:**
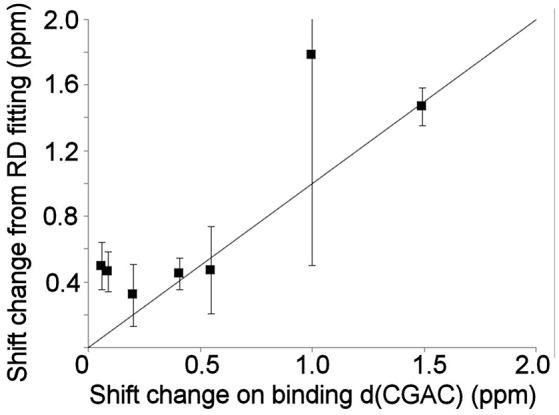
Comparison of the experimental ^15^N chemical shift changes on binding of barnase to d(CGAC) for (L to R) residues 14, 39, 106, 73, 102, 56, and 85, with the chemical shift change obtained by fitting to the relaxation dispersion data for bound barnase. The fit was carried out by fixing the global exchange rate to 500 s^−1^, requiring all residues to have the same forward and back rates, and leaving all other parameters free. The fitted forward rate was 4 s^−1^, as described in the text. The overall correlation coefficient *R*^2^ is 0.62. The errors shown on the fitted shift changes are obtained from the fitting process, and the line has a gradient of 1.

Relaxation dispersion experiments were also carried out on barnase bound to d(CGAC). The results obtained again showed a small number of residues in equilibrium with an excited state (Figure 7). The profiles were smaller for bound barnase than for free barnase, making them harder to fit, nevertheless, all residues fitted to the same exchange rate, within error, suggesting that they all derive from the same conformational exchange process. They were therefore fitted to a common global exchange rate, in a similar way to the fitting procedure for free barnase, giving a global exchange rate of 500 ± 300 s^−1^, and a forward rate of 4 ± 4 s^−1^, corresponding to a population of the excited state of 1% (Figure 7, fitted lines). Significantly, the residues involved were the same as or adjacent to the residues with RD profiles in free barnase (residues 14, 39, 42, 56, 58, 73, 83, 85, 86, 102, and 106), and the chemical shift changes calculated from the relaxation dispersion fitting match well to the experimental shift changes on adding d(CGAC), with an *R*^2^ correlation of 0.62 and a gradient close to 1 (Figure 8). The data for free barnase were shown above to originate from a conformational exchange between 98% free “open” barnase and 2% of a closed state that resembles the bound state. Our data therefore imply that in the presence of excess d(CGAC), bound “closed” barnase is in equilibrium with a small population of a bound “open” state, and that the conformational changes ([free open] ↔ [free closed] and [bound closed] ↔ [bound open]) are similar in both location and magnitude, although in opposite directions. We note that a similar observation was made for ribonuclease A (Loria et al., 2008) and for the closely related protein binase (84% identical to barnase): loop motions were observed to occur in the same place and with the same rate in the absence and presence of inhibitor (Wang et al., 2001). This motion was suggested to be responsible for product release, which is rate limiting for the reaction catalyzed by binase. We cannot make the same conclusion here, because the protein under investigation is a catalytically inactive mutant, and mutations in the active site can have dramatic effects on local fluctuations (Watt et al., 2007; Doucet et al., 2011), although the close similarity of barnase and binase makes it likely that this slow motion in barnase is also related to product release.

### Normal Mode Calculations

**Figure 9:**
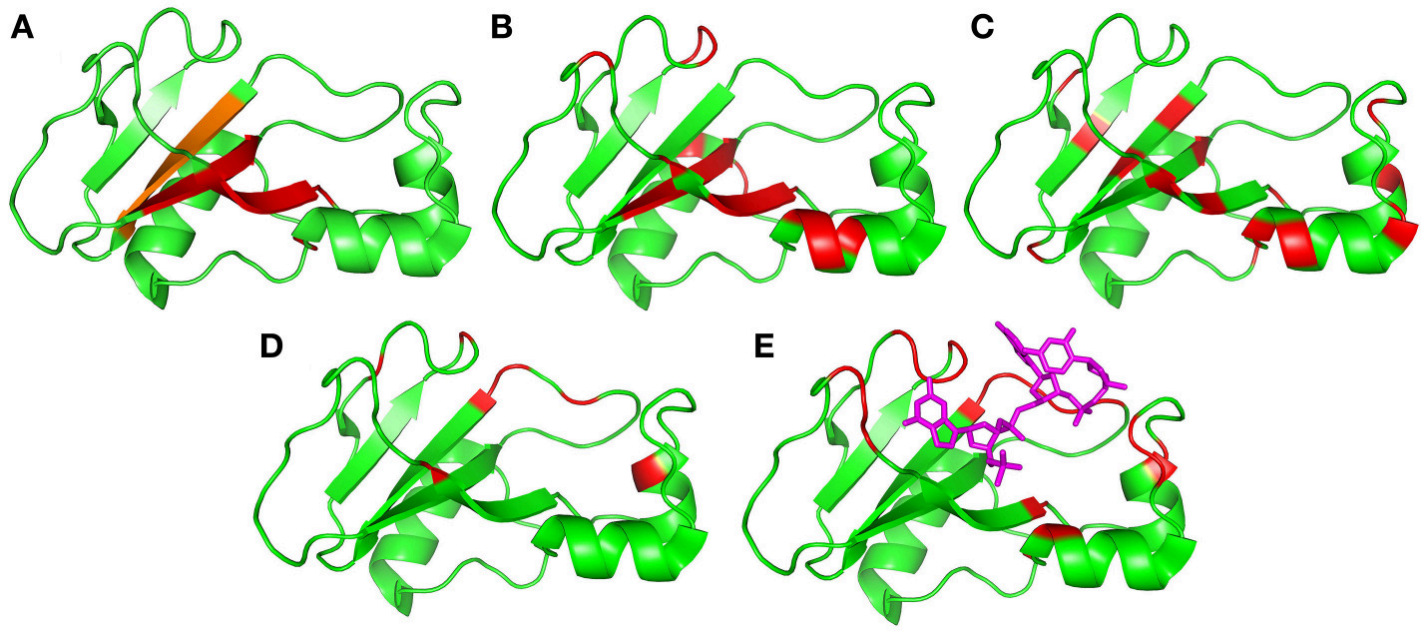
Residues identified as being involved in concerted motions, illustrated using the crystal structure 1brn. Helices are residues 6–18, 26–34, and 41–46, and sheets are residues 51–56, 70–76, 86–92, and 108–109. **(A)** Residues identified as being the major mobile elements in MD/normal mode analysis: 24–25, 50–55, 71–75, and 87–91 (Zhuravleva et al., 2007). The region in orange [87–91] was also identified in an independent analysis of conformational ensembles (Hilser et al., 1998). **(B)** Residues identified in this work from Gdm curvature: 5, 7, 27, 28, 30, 51–54, 60, 71–74, 85, and 100–102. **(C)** Residues previously identified from high-pressure NMR to be involved in sub-μs fluctuations: 23, 26, 28, 34, 40, 44, 50, 53, 55, 75, 87, 89, 93, 97, and 107 (Wilton et al., 2009). **(D)** Residues identified in this work from relaxation dispersion analysis: 36, 39, 42, 43, 56, 58, 83, 85, 86, 101, and 106. **(E)** Residues identified as having large changes in chemical shift and/or methyl group dynamics on binding to barstar: 24, 27, 37, 41, 51, 58–60, 82–86, and 101–104 (Zhuravleva et al., 2007). Shown here for reference is the location of the central two residues of bound d(CGAC) in magenta.

The residues identified by CAPSID as having rapid fluctuations (Figure 9B) are similar to those previously identified by molecular dynamics to form a hinge (Figure 9A). By contrast, the residues identified by relaxation dispersion to have millisecond motions (Figure 9D) are in a different location: they surround the hinge residues, and form a ring or lip around the active site. They are in similar locations to the residues observed to form hydrogen bonds to d(CGAC) in the crystal structure (Buckle and Fersht, 1994), although it is significant that the lip closure observed by relaxation dispersion takes place in the absence of ligand. These observations suggest that the two fluctuation modes are different. In order to investigate this further, we conducted normal mode calculations.

The most obvious computational technique to investigate protein motion is molecular dynamics (MD). MD provides a series of snapshots of how a protein moves, and is thus a comprehensive dataset, but one that contains the local random thermal motions mixed in with the slower collective motions: it is then necessary to extract results of interest from the MD trajectory using statistical tools (e.g., Zhuravleva et al., 2007). By contrast, normal mode calculations directly provide the lowest energy correlated modes. They are fast and simple to analyze, and provide a useful guide to the motions allowed by the ground-state protein architecture, because the calculations are based on a harmonic analysis of the ground-state structure. For this reason, the lowest energy modes are dependent on the details of the starting geometry (van Vlijmen and Karplus, 1999; Batista et al., 2010).

**Figure 10:**
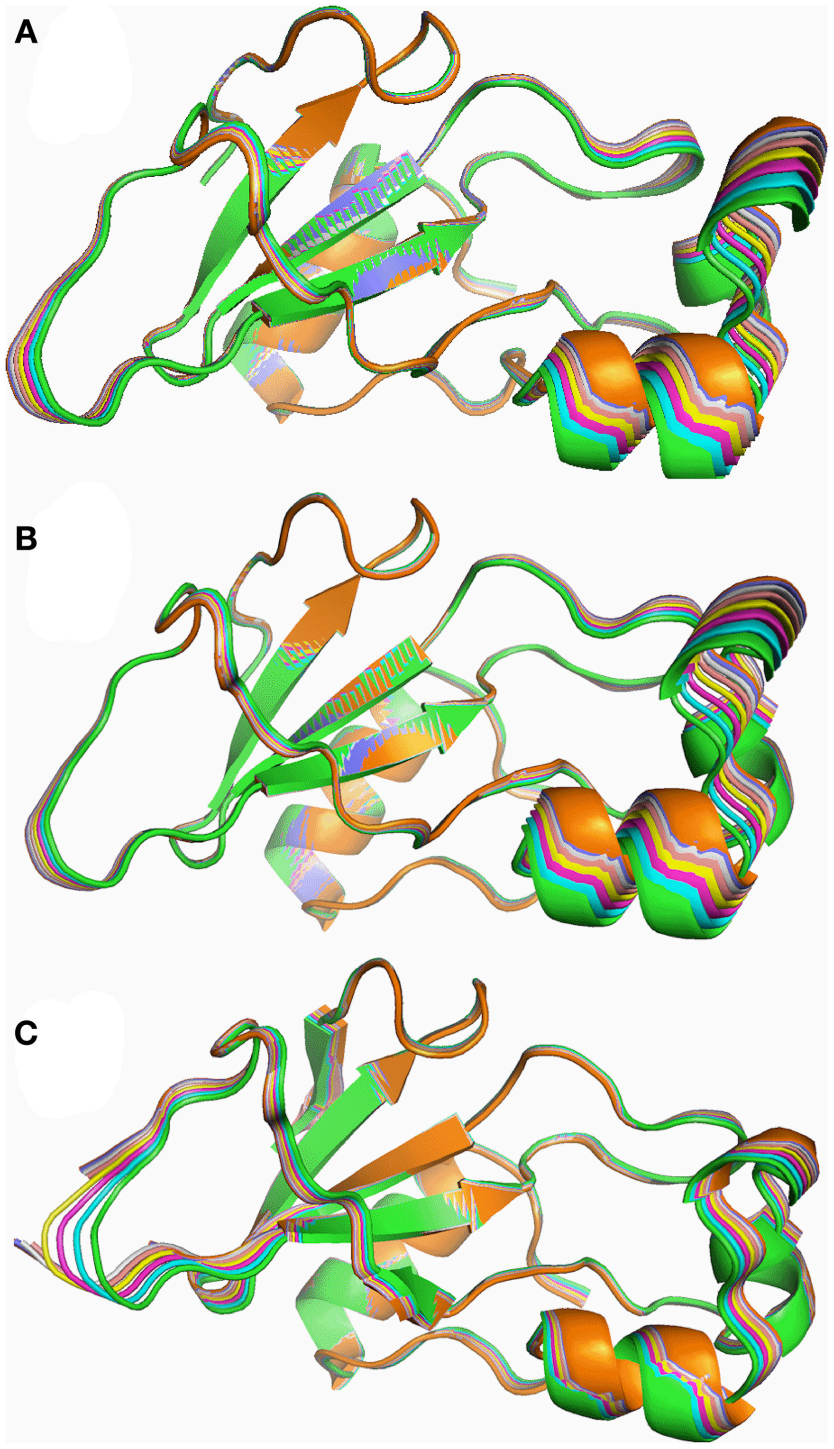
Superposition of 8 structures from the lowest energy normal mode of barnase, corresponding to a 0.5 Å average RMSD, from green (crystal structure) to orange. **(A)** Free barnase (PDB 1a2p). **(B)** Free barnase H102A (PDB 1a2p with the H102 sidechain truncated to CB). **(C)** Bound barnase (PDB 1brn). The next three normal modes look very similar.

Normal mode analysis shows that the six lowest energy modes of free barnase, which together characterize the major low-energy concerted movements in barnase, all involve a bending or twisting of the hinge (Figure 10): residues away from the hinge have increasingly large displacements. Different normal modes involve different symmetric or antisymmetric oscillations around the hinge, while higher modes involve increasingly smaller and less concerted oscillations. The hinge residues lie across the β-sheet, from bottom center to top left in the view shown in Figure 10, and correspond to residues 24–25, 50–55, 71–75, and 87–91 (Figure 9A). A very similar group of residues has been identified in previous computational studies of flexibility in barnase (Hilser et al., 1998; Nolde et al., 2002; Giraldo et al., 2004; Zhuravleva et al., 2007).

Calculations were carried out on free wild-type barnase (Figure 10A) and on the H102A mutant used here for the NMR investigations (Figure 10B). The resultant low-energy modes are almost identical: RMSD values for the lowest energy normal mode of H102A are within 10% of those for the wild type. The normal mode calculations therefore suggest that the mutation does not have a major effect on low-energy fluctuations. The low-energy modes for the bound structure also involve oscillations around the hinge residues (Figure 10C), but in detail the motions are different. For example, the lowest energy mode for free barnase is an in-phase bending of the arms up and down, whereas in bound barnase this motion is the fourth mode, and the lowest mode for bound barnase is more of an out-and-back flex, which is the second mode in free barnase.

**Figure 11:**
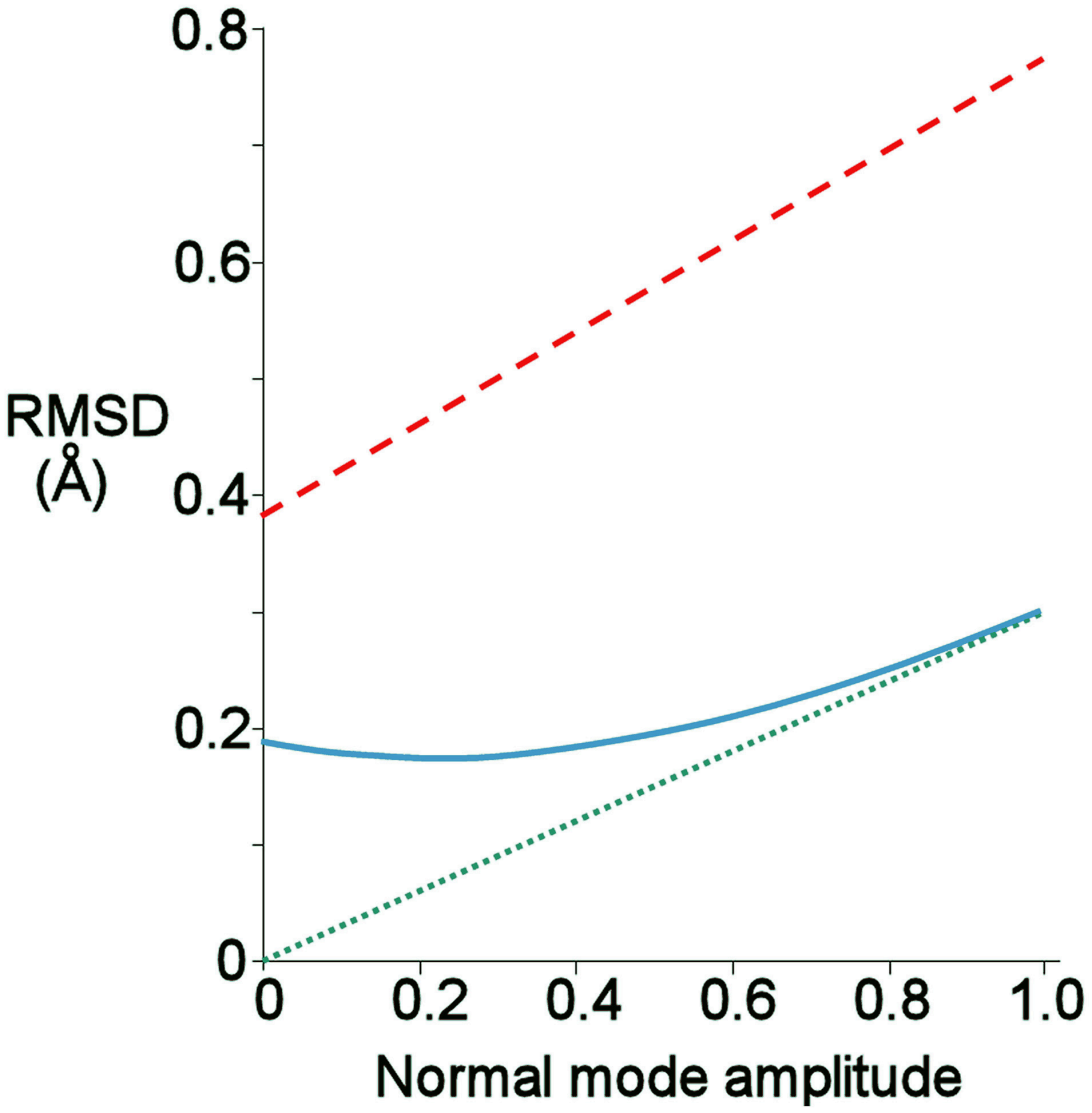
Root-mean-square distance (RMSD) from given starting structure to different points on the lowest energy normal mode trajectory of free barnase, produced using the crystal structure of free barnase (1a2p) as the starting structure. A normal mode amplitude of 1 corresponds to an all-atom RMSD of 0.24 Å. Green: RMSD from 1a2p. Red: RMSD from the closed structure bound to d(CGAC), 1brn. Blue: RMSD from 1brn, but calculated using only Cβ atoms of the residues involved in lip closure (42, 56, 83, 85, 86, 101, and 106).

The normal mode motions (Figure 9A) therefore resemble the fluctuations seen by CAPSID (Figure 9B). By contrast, the motion seen by relaxation dispersion (Figure 9D) resembles the closure of the active site cleft that is seen by X-ray crystallography on ligand binding. In order to compare the two different motions characterized by NMR, we therefore took the normal mode calculation starting from the crystal structure of free barnase (Figure 10A) and compared it both to the “open” crystal structure of free barnase (PDB 1a2p) and to the “closed” crystal structure of barnase bound to d(CGAC) (PDB 1brn). The comparison to 1a2p shows that an increased magnitude of normal mode motion results in a linear increase in the root mean squared distance (RMSD) to the starting structure, as expected (Figure 11, dotted). The comparison to 1brn shows that the free and bound structures differ by 0.39 Å RMSD, and that an increased magnitude of normal mode motion causes the free structure to diverge from the crystal structure of bound barnase, in an almost linear manner with almost the same slope as for free barnase (Figure 11, dashed line). This is not surprising, considering the close similarity of free and bound structures. However, when this analysis is repeated, but now using only the residues involved in lip closure (identified as the residues showing the largest RD profiles for free barnase) (Figure 11, solid line), we see that as the magnitude of the normal motion increases, these residues initially approach each other, and then start to diverge. In other words, the hinge-bending motion of the free protein initially brings the sidechains of the lip residues closer together: the bound conformation is most similar not to the bottom of the energy well for the free protein, but to a conformation higher in energy produced by hinge bending. The hinge bending motion brings the residues lining the lip closer together, and therefore facilitates lip closure.

## Discussion

### Motions Involving More Residues Are Slower (Less Frequent)

Figure 9 summarizes the motions discussed here, and also includes two results from previous studies that have not yet been discussed. Figure 9C shows previous results from our lab, in which the effects of hydrostatic pressure on chemical shifts were used to characterize volume fluctuations in barnase at ambient pressure. The largest volume fluctuations were found in the hinge and surrounding residues, and were significantly reduced on binding of d(CGAC) (Wilton et al., 2009). These fluctuations were estimated to have a timescale of 10^6^–10^9^ s^−1^. Figure 9E presents results from a study of the binding of barnase to its protein inhibitor barstar. Zhuravleva et al. (2007) noted a number of large changes in chemical shift or methyl group dynamics on binding, which are in similar locations to those seen here by RD (residues 24, 27, 37, 41, 51, 58–60, 82–86, and 101–104). They noted that many of these residues are in contact with barstar, and therefore did not attempt to analyze these results further, arguing that the observed changes could result from direct contact with barstar, and therefore cannot be converted directly into conformational information. However, our RD data were obtained in the absence of ligand, and therefore unambiguously show that the protein exchanges with a closed conformation that is different from the ground state, even in the absence of ligand. By extension it is reasonable to argue that many of the effects seen in the previous study (Zhuravleva et al., 2007) may also be due to conformational change rather than the proximity of barstar. We therefore include them in Figure 9.

Mobile regions are shown in Figure 9 arranged in order of number of residues involved, with Figure 9A (normal modes/MD) showing the most localized motion, which in Figure 9B (CAPSID) and Figure 9C (pressure) extends beyond the ends of the β-sheet, and then extends further sideways in Figure 9D (RD) and Figure 9E (ligand binding) to include loop residues. Timescales can also be assigned to most of these motions. All three MD studies (Nolde et al., 2002; Giraldo et al., 2004; Zhuravleva et al., 2007) saw collective hinge bending motions occurring on timescales of a few nanoseconds. Thus, we can expect that the motion shown in Figure 9A will start to appear on a timescale of 10^8^–10^9^ s^−1^. As presented above, CAPSID timescales (Figure 9B) are somewhere in the range 10^6^–10^10^ s^−1^. The residues identified by CAPSID map out a very similar hinge for low-energy collective motion, though rather larger than that observed by MD. We therefore conclude that the motion detected by CAPSID is the same hinge-bending motion. The fluctuations detected by pressure dependence (Wilton et al., 2009) were estimated to have a timescale of 10^6^–10^9^ s^−1^. These timescales can be contrasted with the much slower frequencies observed by RD (Figure 9D), which are 750 ± 200 s^−1^. The timescale associated with substrate binding and release is given by the enzyme turnover rate, which is up to 4,000 s^−1^ depending on the substrate (Day et al., 1992); while the rate of barstar dissociation is 200 s^−1^ at pH 5.8 (Schreiber and Fersht, 1993). Thus, the different motions shown in Figures 9A–E are approximately in order not only spatially but also temporally. These results confirm the simple expectation, that motions that involve a larger number of residues (or bond rotations) are less probable and therefore occur on slower timescales (Henzler-Wildman et al., 2007; Hammes et al., 2011).

It may seem strange to class normal mode calculations in the same group as the MD results as depicting very rapid motions with a rate of 10^8^–10^9^ s^−1^ (a timescale of 1–10 ns). Normal mode calculations are generally described as depicting the lowest energy, and lowest frequency, collective motions, in which case the characteristic motions should be much slower than this. However, motions in proteins are always diffusive rather than harmonic-sometimes described as following Kramers rather than Eyring dynamics, or “overdamped” (Alexandrov et al., 2005)—and thus the normal mode calculations are best thought of as showing how the architecture of a protein allows it most easily to bend, rather than how it actually moves. Experimental studies using neutron spin echo spectroscopy and small-angle neutron scattering (Bu et al., 2005; Inoue et al., 2010), as well as comparisons of MD and normal mode motions (Alexandrov et al., 2005; Swett et al., 2012), have concluded that the interdomain motions described by the lowest frequency normal modes are observed in proteins on timescales of 1–50 ns. In other words, the fast collective motions observed here, on timescales between 10^−10^ and 10^−6^ s, are diffusive rather than harmonic: the protein does not oscillate from one conformation to another, but the residues sample a range of local conformations, some of which conformational changes are correlated and produce hinge bending.

These experimental results demonstrate that the very rapid random thermally induced motions become funneled into increasingly larger scale motions. The differences in timescales are large. The hinge bending (which involves roughly 20 amino acids) occurs ~10^4^ times less frequently than thermally induced rotations about single bonds, implying that the cooperative fluctuation represented by hinge bending only occurs roughly once in 10,000 bond rotations. Similarly, the conformational change of the loops surrounding the active site, which occurs both in the absence and the presence of ligand and represents a conformational change from the open to the closed or “bound” conformation, occurs approximately 10^4^ times slower than the hinge bending, implying that this movement is very rare, occurring only one in 10^4^ hinge bends (or once in 10^8^ bond rotations).

There are two different motional modes. All motions described here have features in common: they all involve hinge bending across the β sheet, and (where measured) each motion has roughly equal magnitude and rate in both free and bound states. However, they can be divided into two groups. The first group (Figures 9A–C) constitutes simple hinge bending, involving an increasingly larger number of amino acids as one goes from Figures 9A–C. There is little if any free energy barrier to this motion, and higher energies/longer timescales just lead to a greater extent of hinge bending being observable. The population of the excited state for CAPSID must be at least 5% to be observable (Williamson, 2003), so this is a well-populated mode. The location, timescale and energy of the motions shown in Figures 8A–C and characterized by MD, normal mode analysis, CAPSID, and high pressure indicate that the motions are different facets of the same fluctuation.

By contrast, the motion observed by RD includes only 1–2% of molecules in the excited state, and occurs at a much slower rate of 750 ± 200 s^−1^: it is thus a much slower and far less likely motion. Furthermore, there is a large free energy barrier between the ground state and excited state, and the excited state is a local energy minimum. The motion is thus a conformational exchange rather than simply a barrierless hinge bending. Finally, the motion also involves an additional set of residues quite distinct from those involved in the hinge-bending motion: these residues are located around the lip of the binding site rather than in the hinge (Figure 9D). The chemical shift changes are in agreement with a conformational selection model, suggesting that the motion seen by RD is an exchange between open and closed forms. The conformational change seen on ligand binding (Figure 9E) involves the same group of residues and occurs at a similar rate, further confirming that the motion is an open/closed equilibrium linked to ligand binding, and is thus a distinct motion from the hinge bending shown in Figures 9A–C.

### The Motional Modes Are Hierarchical

We have characterized two motional modes, but have not yet considered how they are related to each other. In this section, we argue that the two motional modes are hierarchical, and draw out some of the implications. Frauenfelder et al. (2009) describe hierarchical motions within the concept of an energy landscape. They describe the energy landscape divided into several tiers, distinguished by the rate at which conformations can cross from one basin into the next. The highest tier (tier 0) has free energy barriers that take times of the order of micro- to milli-seconds to cross; within each energy basin lie many tier-1 substates that exchange much more rapidly. These two exchange processes can therefore be described as hierarchical, provided of course that they both involve the same energy basin. Henzler-Wildman et al. (Henzler-Wildman and Kern, 2007; Henzler-Wildman et al., 2007) use the same concepts, arguing that exchanges of states within tier 1 are characteristically collective motions of loops and can occur within nanoseconds, while exchanges of states within tier 2 are typically sidechain rotations and occur within picoseconds. They also stress that (in their system) large-amplitude rapid fluctuations occur in the same locations as large-amplitude slow fluctuations, and suggest that “the physical origin of the catalytically important collective domain motions (microseconds to milliseconds) is the fast-timescale local hinge motions” (Henzler-Wildman and Kern, 2007), leaving open the question of the physical mechanism for linking these processes together. For barnase, we can now describe what this physical mechanism is.

**Figure 12:**
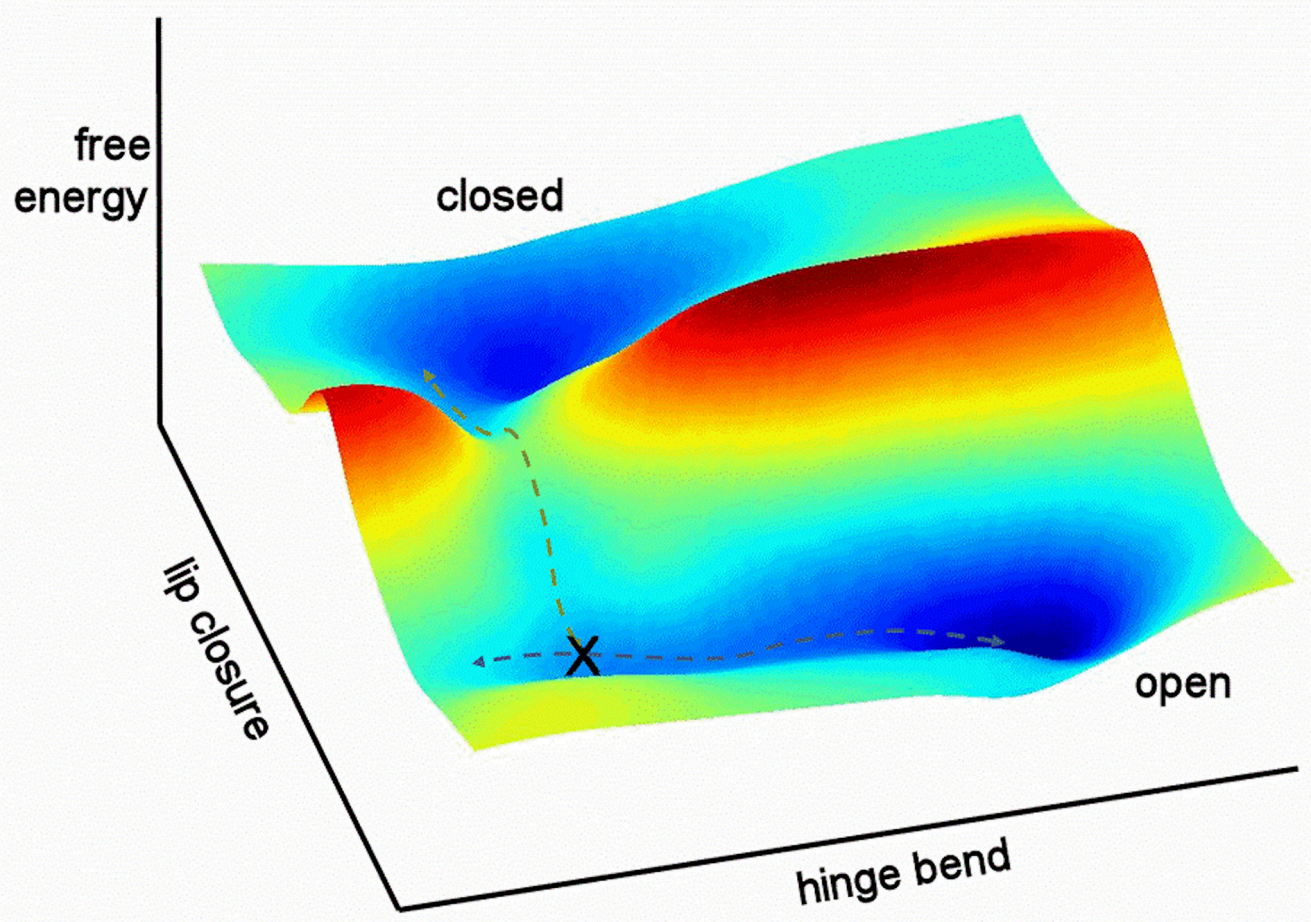
Relationship between the two fluctuations discussed here. Hinge bending has no free energy barrier to fluctuation: higher energy states merely occupy a larger conformational space. By contrast, lip closure passes over a high-energy transition state. The two pathways overlap at position X.

It is helpful to sketch the general features of the energy landscape for free barnase as characterized here (Figure 12). In this figure, the multidimensional collective reaction coordinate from open to closed (Hammes-Schiffer and Benkovic, 2006) is compressed into two dimensions, comprising a hinge bend and a lip closure. Free barnase occupies the “open” basin 98% of the time. This basin allows low-energy hinge bending on a timescale of the order of 10^−9^–10^−7^ s, with ~5% of molecules in the vicinity of the conformation denoted X in Figure 12. Conformational exchange within the “open” basin therefore corresponds to Tier-1 fluctuations in Frauenfelder's description (Frauenfelder et al., 2009), and is dominated by hinge-bending, a mode that is dictated by the architecture of the protein. Barnase is also able to exchange over a large free energy barrier into the “closed” energy basin, a Tier-0 fluctuation (and the only Tier-0 fluctuation detected here for free barnase). This exchange is much slower, taking place at a rate of 750 ± 200 s^−1^. The normal mode calculations (Figure 10) suggest that this exchange takes place not from the bottom of the energy well but from a partially hinge bent position part way up the slope leading up from the bottom of the well: the position denoted by X in Figure 12.

Any molecule that progresses from the open to the closed basin is very likely to get there via the low-energy path described in Figure 12: it undergoes hinge bending to produce a conformation resembling X, and then undergoes lip closure. Any other route is less probable and therefore of higher energy. In other words, the two fluctuations share a common set of conformations (X). This is a necessary feature of hierarchical fluctuations, and implies that the two fluctuations must to some extent be colocalized (McDonald et al., 2013). It is not necessary for the amplitudes of motion to be common to the two modes (Henzler-Wildman and Kern, 2007), and indeed in barnase they are not. The importance of the rapid hinge-bending fluctuation is that it generates a population of molecules with an appropriate degree of hinge bending to allow ready lip closure. This is the physical basis for hierarchical motion: the faster motion acts as a “promoting motion” (Agarwal et al., 2002; Loveridge et al., 2011) to produce a suitable conformation from which the slower motion can begin. In turn, the slower motion produces a closed conformation that is presumably better organized to promote catalysis. Thus, the role of these motions is to enable the enzyme to efficiently attain the conformation that best catalyzes the reaction (Pisliakov et al., 2009; Antoniou and Schwartz, 2011; Loveridge et al., 2011; Doshi et al., 2012). The physical connection between the two fluctuations (i.e., via conformation X) establishes that the route from open to closed is not a consequence of two fluctuations in parallel (Hammes et al., 2011) but two sequential fluctuations, with the slower fluctuation dependent on the faster. The argument presented here is necessarily a gross simplification, because it has compressed a complicated multidimensional fluctuation into only two dimensions, and therefore smoothed out many of the energy barriers. The Frauenfelder tier categorization is simple but so far seems to correspond remarkably well to a wide range of experimental observations.

The model proposed here is different in detail from some existing models. Agarwal (Agarwal, 2006; Ramanathan and Agarwal, 2011) and others (Tousignant and Pelletier, 2004), in a development of Frauenfelder's arguments (Fenimore et al., 2004), propose that solvent fluctuations are coupled to fluctuations in flexible loops, which in turn are coupled to the active site via networks incorporating critical hydrogen bonds. In our model the coupling is more diffuse in that it uses architectural features of the protein to channel motions into specific functionally important modes. It is thus much more similar to the segmented transition pathway proposed for NtrC (Lei et al., 2009), which involves four different fluctuations, possibly assisted by transient hydrogen bonds.

The hierarchical relationship between the two fluctuations does not imply that one motion “causes” the other (Benkovic et al., 2008), but merely that the faster provides the platform from which the slower can start, by increasing the population of molecules with conformations similar to X. We note again that “faster” means more frequent: the speed at which individual atoms move is not changed, and arises from thermal motion. There is therefore no contradiction in having a slower motion dependent on a faster one. It seems likely that proteins are not at thermal equilibrium over functional timescales (Hu et al., 2016). This would further emphasize the importance of the *pathway* rather than just the overall *energetics*.

All the discussion so far has been based on the dynamic energy landscape for the free protein. The limited evidence available suggests that the landscape for the bound protein has a similar overall shape, with minima and maxima in similar places, although the bound state energy well is deeper, and the free state well is shallower. Binding of the ligand produces an energy bias in the landscape, such that more lip-closed conformations are lower in energy, without effecting a big change in the shape. One can easily imagine that the detailed route from open/free to closed/bound will be more complex than that shown in Figure 12, and likely involve a coupling between binding and closure, or between induced fit and conformational selection (Arora and Brooks, 2007). The route from closed to open is not necessarily the same in reverse, although the similarities of free and bound landscapes suggests that it will be similar.

The relationship between the two fluctuations discussed here implies that it might be possible to identify mutations in which one motion is affected but not the other, as discussed in Gagné et al. (2016). Such investigations are outside the scope of this work.

It is an interesting question whether the results of this study can be extended to other proteins. We believe that they can. This claim is based on two main arguments, of which the first is that the general features of the energy landscape discussed here are common to many proteins, not just barnase. For all enzymes, catalytic efficiency is an evolved process. Commonly the starting point is a poorly functional *architecture*, to which optimal active site structure can be added (Williamson, 2011). This matches a key conclusion here, that it is the architecture of the protein that determines the allowable large-scale motions, and which funnels random thermal motion into catalytically useful motion. However, closure around the active site to create the reaction-ready conformation is much more specific to the enzyme, and is thus a different type of conformational change. The second argument is that similar features have been identified in most enzymes that have been studied in detail, as described in the introduction. One further example is pertinent, namely β-phosphoglucomutase (PGM), which catalyzes the interconversion of β-glucose 1-phosphate into glucose 6-phosphate via β-glucose 1,6-bisphosphate. A recent study (Johnson et al., 2018) has shown that the enzyme undergoes a typical domain closure on binding substrate, which brings the nucleophilic oxygen within van der Waals contact of the transferring phosphorus. However, this conformation is not yet catalytically active: it requires a second conformational change localized to a loop containing T16 and D10 to produce a conformation capable of catalysis. We therefore propose that hierarchical motions will prove to be typical of many, even most, enzymes.

## Conclusions

Barnase undergoes two collective motions: hinge bending, which is a smooth fluctuation without significant free energy barriers, and lip closure, which is a slow conformational exchange with a large free energy barrier. These act as promoting motions (Agarwal et al., 2002; Loveridge et al., 2011) to produce a reaction-ready state. These motions are hierarchical (Henzler-Wildman et al., 2007; Frauenfelder et al., 2009), in the sense that lip closure happens from a partially hinge-bent state. The structure of the enzyme is such that hinge bending is a low energy distortion of the ground state, and lip closure is a facile collective mode, but only from a partially hinge-closed conformation. Such a general mechanism could be very common in a range of protein and enzyme interactions (Tobi and Bahar, 2005; Hammes-Schiffer and Benkovic, 2006; Henzler-Wildman et al., 2007), not least because such a two-step pathway is straightforward to optimize by evolution (Klinman and Kohen, 2014). We note that the guanidinium-dependent temperature coefficient technique is useful for characterizing motions at timescales around 10^8^ s^−1^, an important timescale that is difficult to access by other methods (Lange et al., 2008; Wilton et al., 2009).

## Author Contributions

MW designed experiments. MP, SS, AH, and NB prepared materials and collected data. MP, NB, and MW analyzed data and wrote the paper. All authors approved the final version of the manuscript.

### Conflict of Interest Statement

The authors declare that the research was conducted in the absence of any commercial or financial relationships that could be construed as a potential conflict of interest.

## Abbreviations

MD: molecular dynamics
RD: relaxation dispersion
CPMG: Carr-Purcell-Meiboom-Gill
CAPSID: Curvature of amide proton shifts induced by denaturant
HSQC: Heteronuclear single-quantum correlation spectroscopy
PDB: protein data bank
RMSD: root mean square deviation.
